# Backward Acoustic Waves in Piezoelectric Plates: Possible Application as Base for Liquid Sensors

**DOI:** 10.3390/s23020648

**Published:** 2023-01-06

**Authors:** Andrey Smirnov, Boris Zaitsev, Ilya Nedospasov, Gleb Nazarov, Iren Kuznetsova

**Affiliations:** 1 Kotelnikov Institute of Radio Engineering and Electronics of RAS, Moscow 125009, Russia; 2 Kotelnikov Institute of Radio Engineering and Electronics of RAS, Saratov Branch, Saratov 410019, Russia

**Keywords:** backward plate acoustic waves, detection, dispersion curves, interdigital transducer, contact with liquid, zero group velocity

## Abstract

Backward acoustic waves are characterized by oppositely directed phase and group velocities. These waves can exist in isotropic and piezoelectric plates. They can be detected using a set of interdigital transducers with different spatial periods located on the same piezoelectric substrate. In this paper, the effect of a nonviscous and nonconductive liquid on the characteristics of a first-order backward antisymmetric wave in a YX plate of lithium niobate is studied theoretically and experimentally. It is shown that the presence of liquid does not lead to the transformation or disappearance of this wave. It is shown that these waves are close to the cutoff frequency and are characterized by the presence of a point with zero group velocity. The design of a liquid sensor based on these waves is proposed.

## 1. Introduction

In recent years, the study of backward acoustic waves in geometrically bounded media has been actively carried out. Backward waves have oppositely directed phase and group velocities, i.e., the energy flux velocity of these waves is negative [1,2,3,4,5,6,7]. The possibility of the existence of such waves was shown for isotropic plates in the middle of the last century [8,9]. The characteristics of backward acoustic waves in multilayer structures [1,10,11], phonon crystals [12,13], pipes, shells and hollow cylinders, soft ribbons, and functionally graded piezoelectric-piezomagnetic materials [14,15,16,17,18,19,20,21] are currently being studied. It was shown that in the elastic plates, the backward acoustic waves can be polarized both in the sagittal plane (Lamb waves) and in the shear-horizontal direction (SH waves) [22,23,24]. The backward acoustic waves of higher orders in isotropic plates and multilayered structures were also considered in [25,26,27,28]. Active studies of backward acoustic waves in piezoelectric plates and structures with large piezoelectric constants (potassium niobate, lithium niobate, SOI/AlN) have been carried out recently [6,7,24,29,30]. It has been stated that the reason for the appearance of backward Lamb waves is the proximity to each other of the frequencies of thickness resonances corresponding to bulk acoustic waves (BAW) of different polarizations [2]. As for the backward waves with shear-horizontal polarization, they can exist only in the piezoelectric plates. Their appearance can be explained by the presence of local concavity of the section of slowness surface for the shear BAW in the propagation direction of the backward SH wave [6]. Devices based on the negative refraction of elastic-guided waves were suggested recently [31]. It was also theoretically shown that the properties of these waves depend on the electrical boundary conditions on the surface of the piezoelectric plate [32]. A method for the detection of backward acoustic waves in piezoelectric plates using a set of interdigital transducers with different wavelengths was developed recently [29,33]. The possibility to control the type of wave excited by using a dual-array transducer or acoustic meta-surface was suggested in [34,35].

Interest in backward waves is caused, among other things, by the possibility of using them to create high-quality acoustic resonators [3]. This principle is based on the possibility of using a certain resonator frequency at which a wave with zero group velocity is excited. In this case, the energy of the wave will be concentrated in the plate in the region of the exciting transducer, which will lead to an increase in the Q factor of such a developed resonator. Obviously, such a resonator will be super-sensitive to changes in the properties of the environment. On this basis, it will make it possible to develop a new class of acoustic sensors. However, for the realization of such devices, it is necessary to provide space-time synchronism with great accuracy. This is due to the fact that the frequency range of the existence of the backward waves is rather small, and it is necessary to have the possibility of fine-tuning the frequency for a fixed value of the plate thickness.

Another aspect of the investigation of the properties of backward acoustic waves is the study of the influence of liquid on their properties. The backward waves in isotropic plates immersed in a liquid were studied both theoretically and experimentally [36,37,38,39,40,41]. It has been shown that due to the complexity of the wave number of the backward acoustic waves, as well as the leaky waves, the group and the energy flux velocities are not equal. The new methods based on the analysis of the energy fluxes of the leaky waves in a plate and liquid [39,42], as well as an analysis of the phase shift of the transmitted wave through a plate immersed in a liquid [43], were developed.

Earlier, a set of interdigital transducers (IDTs) with different wavelengths located on the same plate was proposed for the experimental registration of backward acoustic waves [29,33]. However, the possibility of backward wave detection in a plate in contact with a liquid by this method has not been previously demonstrated.

Thus, in this work, a theoretical analysis of the propagation of backward acoustic waves in a piezoelectric plate in contact with a non-viscous and non-conductive liquid was carried out. Based on the analysis performed, the corresponding periods of the IDT were chosen. This set of IDTs made it possible to pass through the region of the existence of backward acoustic waves. An experiment was carried out to register the effect of liquid on backward acoustic waves, and the design of the corresponding liquid sensor was proposed.

## 2. Materials and Methods

### 2.1. Theoretical Boundary Transfer Matrix Method

**Figure 1:**
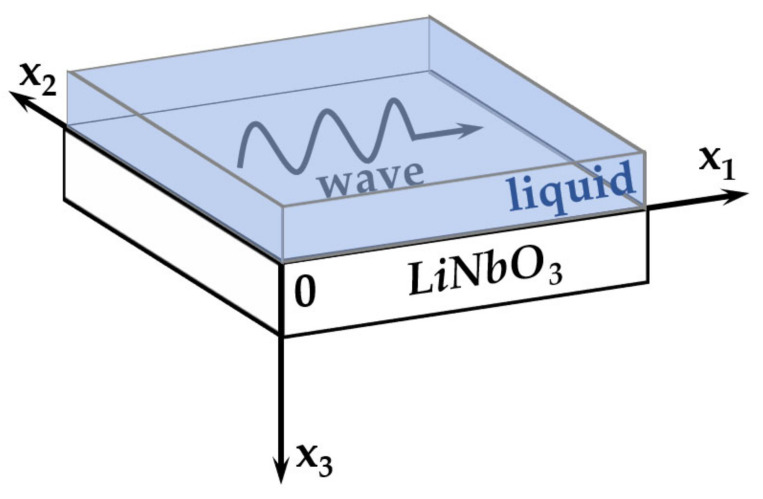
Geometry of the problem.

The dispersion dependences of the forward and backward acoustic waves in a piezoelectric plate contacted with liquid were obtained during the consideration of the following problem. The geometry of the task is presented in Figure 1. The wave propagates along the axis *x_1_* of the plate bounded by the planes *x_3_* = 0 and *x_3_* = h. The region *x_3_* < 0 is occupied by distilled water and region *x_3_* > h corresponds to vacuum. We consider it a two-dimensional problem, so all mechanical and electrical variables are assumed to be constant along the *x_2_* axis.

The motion equation, Laplace’s equation, and constitutive equations for the piezoelectric medium have the following form [44]:

(1) $$\rho \partial^{2}U_{i}/\partial t^{2}=\partial T_{ij}/\partial x_{j},\;\;\partial D_{j}/\partial x_{j}=0,$$

(2) $$T_{ij}=C_{ijkl}\partial U_{l}/\partial x_{k}+e_{kij}\partial \Phi /\partial x_{k},\;D_{j}=-\varepsilon_{jk}\partial \Phi /\partial x_{k}+e_{ijk}\partial U_{l}/\partial x_{k}.$$

Here *U_i_* is the component of the mechanical displacement of the particles, *t* is the time, *T_ij_* is the component of the mechanical stress, *x_j_* is the coordinate, *D_j_* is the component of the electrical displacement, *Φ* is the electrical potential, *ρ*, $C_{ijkl}$, $e_{ikl}$, and $\varepsilon_{jk}$ are the density, elastic, piezoelectric, and dielectric constants, respectively.

In the region *x_3_* > *h*, the electrical displacement must satisfy Laplace’s equation:

(3) $$\partial D_{j}^{V}/\partial x_{j}=0,$$

where $D_{j}^{V}=-\varepsilon_{0}\partial \Phi^{V}/\partial x_{j}$. Here index *V* denotes the values referred to the vacuum and *ε*_0_ is the vacuum permittivity.

The presence of a non-conductive, non-viscous liquid in the region *x_3_* < 0 could be described by additional equations such as a motion equation, Laplace’s equation, and corresponding constitutive equations [45]:

(4) $$\rho^{lq}\partial^{2}U_{i}^{lq}/\partial t^{2}=\partial T_{ij}^{lq}/\partial x_{j},\;\;\partial D_{j}^{lq}/\partial x_{j}=0,$$

(5) $$T_{ij}^{lq}=C_{ijkl}^{lq}\partial U_{l}^{lq}/\partial x_{k},\;D_{j}^{lq}=-\varepsilon_{jk}^{lq}\partial \Phi^{lq}/\partial x_{k}.$$

Here the superscript “*lq*” corresponds to liquid.

The acoustic wave propagating in such a structure should also satisfy the mechanical and electrical boundary conditions. At the plane *x_3_* = 0, these conditions have the next form:

(6) $$U_{3}^{lq}=U_{3};\;T_{33}^{lq}=T_{33};\;T_{13}=T_{23}=0;\;\Phi^{lq}=\Phi ;\;D_{3}^{lq}=D_{3}.$$

At the plane *x_3_* = *h* ones have the following form:

(7) $$T_{3j}=0;\;\Phi^{V}=\Phi ;\;D_{3}^{V}=D_{3}.$$

The solution to the boundary problem described above can be represented as a set of planar inhomogeneous waves [45]:

(8) $$Y_{i}\left(x_{1},x_{3},t\right)=Y_{i}\left(x_{3}\right)e^{j\omega \left[t-x_{1}/V_{ph}\right]},$$

where *i* = 1–8 for the piezoelectric plate, and *i* = 1, 2 for vacuum, mechanical and electrical parts of the task for liquid, *V_ph_* is the phase velocity. Here the following normalized values were introduced:

(9) $$Y_{i}=\omega C_{11}^{*}U_{i}/V_{ph};Y_{4}=T_{13};\;Y_{5}=T_{23};Y_{6}=T_{33};\;Y_{7}=\omega e^{*}\Phi /V_{ph};\;Y_{8}=e^{*}D_{3}/\varepsilon_{11}^{*},$$

where *i* = 1–3, $C_{11}^{*}$, $\varepsilon_{11}^{*}$ are the normalizing material constants of the piezoelectric medium in the crystallographic coordinate system, e* = 1 and it has the dimension of the piezoelectric constant.

The substitution of (8) into Equations (1)–(5) yields one system of 8 and three systems of 2 ordinary differential equations for the piezoelectric medium, vacuum, mechanical and electrical parts of the task for liquid, respectively. Each of these systems can be written in the following matrix form:

[*A*][d*Y*/d*x_3_*] = [*B*][*Y*] (10)

Here [d*Y*/d*x_3_*] and [*Y*] are the eight-dimensional vectors for the piezoelectric media, and three two-dimensional vectors for the vacuum, mechanical and electrical parts of the task for liquid, respectively. The components of these vectors are determined in accordance with the expressions (9). The appropriate parameters of the piezoelectric media were taken for obtaining the *Y_i_* normalized values for liquid. The matrices [*A*] and [*B*] are square with the dimension of 8 × 8 for piezoelectric medium, and three of 2 × 2 for vacuum, mechanical and electrical parts of the task for liquid. Because the matrix [*A*] is singular (det[*A*] ≠ 0) one can write for each medium [d*Y*/d*x_3_*] = [*A*^−1^][*B*][*Y*] = [*C*][*Y*].

Further, to solve the system of Equation (10), it is necessary to find the eigenvalues *β*(*i*) of the matrix [*C*] and corresponding eigenvectors [*Y*(*i*)], which determine the parameters of the partial waves for each medium. The general solution is a linear combination of all partial waves for each medium:

(11) $$Y_{k}=\overset{N}{\underset{i=1}{\sum }}A_{i}Y_{k}^{\left(i\right)}e^{\beta_{i}x_{3}}e^{j\omega \left[t-x_{1}/V_{ph}\right]},$$

where the numbers of the eigenvalues *N* = 8 for the piezoelectric medium, and *N* = 2 for the vacuum, mechanical and electrical parts of the task for liquid, *A*_i_ are the unknown values. For the determination of the values *A*_i_ and velocity *V_ph_*, the mechanical and electrical boundary conditions (6) and (7) were used. These conditions were presented in the normalized form taking into account (9). Due to the piezoelectric plate being bounded, all of the eight eigenvalues of the corresponding matrix [*C*] were taken into account. The eigenvalues of the matrixes [*C*] that correspond to mechanical and electric variables for liquid in the region *x*_3_ < 0 and have positive real parts were excluded from consideration. As vacuum is in the region *x*_3_ > *h* and all its electric variables should have amplitudes decreasing with distance from the plate, the eigenvalues of the corresponding matrix [*C*] with a negative real part were excluded from consideration.

Thus, the unknown quantities *A_i_* and the velocity *V_ph_* for each type of wave can be determined from the systems of 11 homogeneous algebraic linear Equations (6) and (7).

### 2.2. Experimental Study

An experimental sample with 11 interdigital transducers (IDTs) was produced to confirm the theoretical results. The IDT wavelength was varied in the range from 1.0 to 2.0 mm with a step of 0.1 mm. Each IDT contained 5 pairs of fingers and had an aperture of 9 mm. A *Y*-cut lithium niobate plate polished on both sides with a thickness of 320 μm was used as a substrate.

IDTs were produced by maskless projection photolithography. Initially, the wafer was pre-cleaned by means of washing with acetone, isopropyl alcohol, and ionic cleaning in argon plasma. Then, an aluminum coating with a thickness of 400 nm was deposited on the surface of the plate by magnetron sputtering at the discharge of 250 W, deposition time of 3 min, and pressure in the chamber of 5.6 × 10^−3^ Torr. Next, a S1813SP15 photoresist (Shipley, Sasagami, Japan) of 2 μm thick was applied to the aluminum surface using a centrifuge. After that, the photoresist was tanned for 30 min at a temperature of 94 °C.

**Figure 2:**
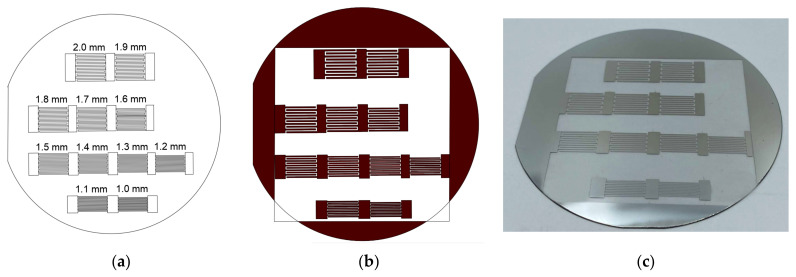
(**a**) Schematic view of the location of the IDTs on a Y-cut lithium niobate wafer; (**b**) the location of the photomask on the plate; (**c**) photo of the manufactured experimental sample.

Photolithography was carried out using a SmartPrint setup (Microlight 3D, Grenoble, France). The location of the IDTs and photomask on the plate is shown in Figure 2a,b, respectively. The exposed part of the photoresist was removed using a P-236A-MF metal-free developer (FRAST-M, Moscow, Russia). The part of the aluminum coating not protected by the photoresist was removed with a mixture of orthophosphoric and nitric acids (95:5). Figure 2c shows a photo of the manufactured experimental sample.

**Figure 3:**
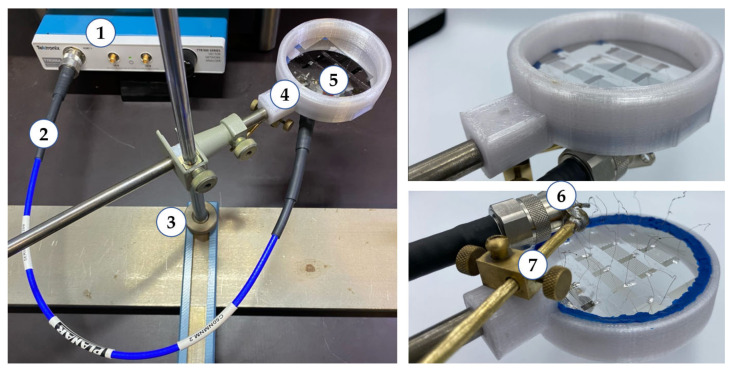
The experimental setup: (1) vector network analyzer, (2) phase-stable cable assembly, (3) fixing post, (4) piezoelectric plate holder, (5) YX LiNbO_3_ plate with system of IDTs, and (6) switch connector holder.

A photo of the experimental setup used is shown in Figure 3. A Tektronix TTR506 vector network analyzer (Beaverton, OR, USA) (1) was connected to an N-type connector (6) using a phase-stable cable assembly (2). The plate with IDTs (5) was fixed in the sample holder (4). This holder was modeled and printed using a VolgoBot A4 PRO2.8 extrusion 3D printer (Volgobot, Volgograd, Russia). The sides of the holder (4) served as a cell for water. The electrode structures were located on the underside of the plate. Plasticine was used to fix the plate in the holder. It also served as a sealant to protect the IDT pads from liquid and prevented wave re-reflections from the plate boundaries. In the space between the transducers, a viscous water-soluble polymer material based on polyethylene oxide was applied for damping. The IDT was connected to a connector (6) using thin copper wires 20 mm long and soldered with indium. The stand (3) made it possible to firmly fix the holder pin (4) during the measurement. The bronze holder (7) made it possible to position the connector opposite a certain IDT and to minimize the length of the contact wires.

The measurements were carried out as follows. The connector (6) was positioned opposite the required transducer with the help of a movable holder (7). Then the contact wires from the IDT were soldered to the connector (6) and the parameter S11 was measured in a given frequency range. After that, distilled water was set into the cell, and the S11 parameter was measured in the presence of liquid. Similar measurements were carried out for each transducer. The height of the guide fixing post (3) was 50 cm. Thus, the experimental sample was fixed at a height of 45 cm from the table. This made it possible to gain access to the underside of the plate without changing the position of the holder (5) with the sample (6). The vector network analyzer was calibrated using OSLT compact calibration kit (4-in-1) 0–9 GHz N male, Spinner BN 533884 (Spinner, Munich, Germany).

## 3. Results and Discussion

### 3.1. Theoretical Results

**Figure 4:**
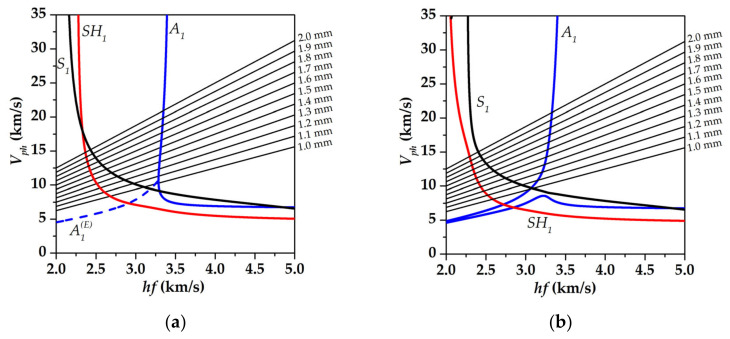
The dispersion curves of S_1_, SH_1,_ and A_1_ acoustic waves in (**a**) free YX *LiNbO_3_* plate and in (**b**) structure “YX *LiNbO_3_* plate—distilled water”. The straight solid lines are auxiliary lines *V_ph_* = *λf* (*λ* is wavelength).

As a result of the calculations, the dispersion curves of the phase velocities of the acoustic waves in the YX *LiNbO_3_* plate with/without distilled water on the surface in the range of the parameter *hf* of 2–5 km/s were obtained (*h* is plate thickness, *f* is the wave frequency). The material constants for lithium niobate taken from [46] are presented in Table 1. The density *ρ^lq^*, permittivity *ε^lq^*, and elastic constant *C^lq^* of distilled water were equal to 997.299 kg/m^3^, 80, and 2.25 GPa, respectively. As it has been shown earlier for YX *LiNbO_3_* in the range *hf* = 2.5–4 km/s there are only two piezoelectric waves a shear—horizontal SH_1_ wave and antisymmetric A_1_ Lamb wave [29,30,47]. Figure 4 shows the dispersion curves for these waves for free YX *LiNbO_3_* plate and at contact with distilled water. It has been found that for both cases the dispersion dependence for the A_1_ wave has both the forward and backward branches.

As it has been shown in [30,33] for registration of backward acoustic waves it is possible to use a set of IDT with various spatial periods. In order to determine the needed values of the spatial period (wavelength) *λ* of the IDTs, the auxiliary lines *V_ph_ = λf* for different values of *λ* were used (Figure 4). The values of the spatial period of the IDTs obtained in such a way allowed us to observe the transition from the forward wave region of the dependence to the backward one. The analysis of Figure 4a has shown that the growth of the IDTs period should lead to monotonically decreasing the resonant frequency of the SH_1_ wave at a fixed plate thickness (*h* = 0.49 mm). As for the A_1_ wave, there exists two regions of the dispersion curve (blue color) corresponding to forward and backward waves. The point of the transition from one type of wave to another is called “point of zero-group velocity” (ZGV). In the region corresponding to the forward wave, the wave frequency should decrease at the IDTs period increase. After the ZGV point when the dispersion dependence passes to the backward wave region, the resonant frequency should increase.

An analysis of Figure 4b shows that the presence of liquid on the plate surface leads to a pushing apart between the forward and backward branches of the dispersion curve of the A_1_ wave, as well as to a broadening of the frequency range of the existence of backward leakage waves [48]. It should be noted that in the absence of liquid (Figure 4a), there are two complex conjugate solutions in the considered plate, with the same values for the real part of the phase velocity, corresponding to the evanescent ${A_{1}}^{E}$ waves [33]. The imaginary parts of the phase velocities of these waves differ in sign. These two waves cease to be evanescent and the real parts of their phase velocities become different at a load appearance on the plate surface. At the same time, these waves remain backward. One can also see the appearance of pushing apart between the dispersion dependences for the S_1_ and SH_1_ waves due to the appearance of a liquid load on the plate surface. These effects are associated with a change in the boundary conditions on the surface of the plate, and, accordingly, with a change in the resonant characteristics of the structure under consideration. It is necessary to note that the phase velocity of the acoustic waves does not change significantly due to the presence of non-viscous and nonconductive liquid. So we can use the same set of IDTs for cases of the absence and presence of distilled water on the plate surface. As has been shown earlier [29], the S_1_ wave is non-piezoactive for a given crystallographic orientation. This is one reason why it is not expected to be detected during experiments.

### 3.2. Experimental Results

**Figure 5:**
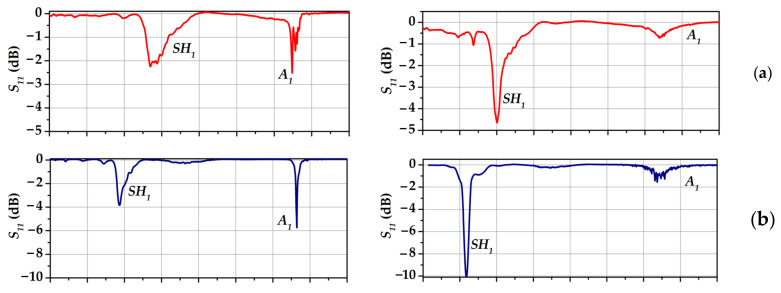

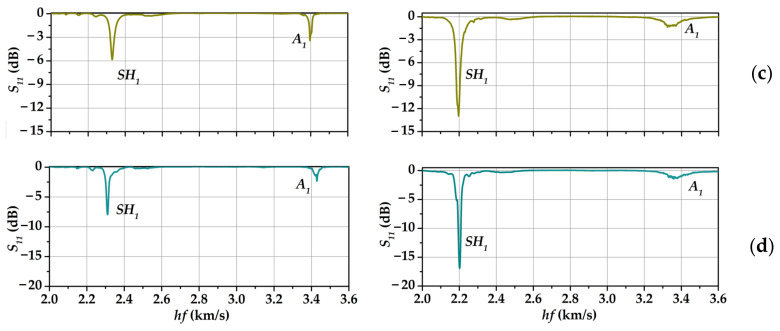
The frequency dependences of *S_11_* parameters of IDTs with *λ* of (**a**) 1.0 mm, (**b**) 1.4 mm, (**c**) 1.8 mm, and (**d**) 2.0 mm. The left and right columns correspond to contact with air and liquid, respectively.

The results of measurements of the *S_11_* parameter of acoustic waves propagating in the YX *LiNbO_3_* plate in the absence (left column) and presence (right column) of an inviscid non-conductive liquid are shown in Figure 5.

An analysis of the results obtained has shown that in the frequency range of 3–8 MHz, there are two acoustic waves of the first order and these are shear-horizontal SH_1_ and antisymmetric A_1_ waves [29,30,32]. It is possible to see that the presence of a non-viscous and non-conductive liquid on the surface of a piezoelectric plate does not change the frequency of the backward acoustic wave but significantly reduces its amplitude. This is attributed to the type of backward wave, i.e., it is an antisymmetric wave of the first order. Its maximum mechanical displacement component is the component perpendicular to the plate, which leads to a significant decrease in its amplitude due to the emission of acoustic energy into the liquid.

**Figure 6:**
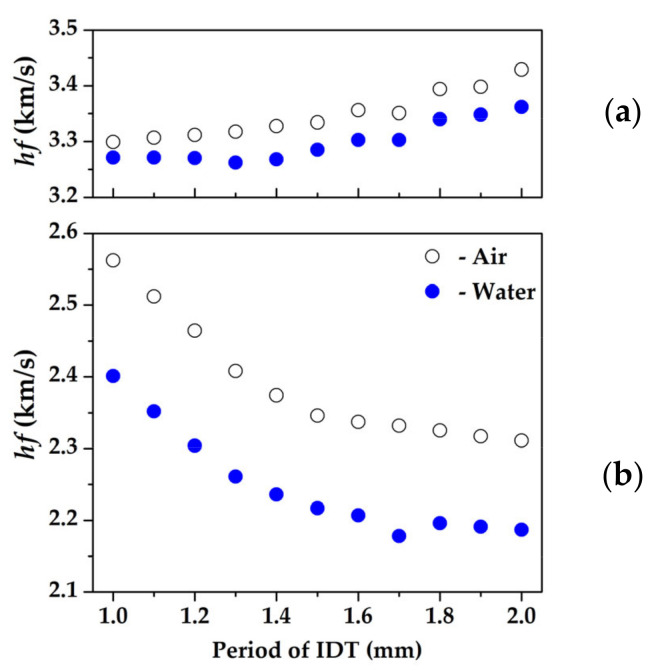
Dependences of resonant frequencies of (**a**) A_1_ and (**b**) SH_1_ acoustic waves on the IDT period in the contact with air and water.

The dependences of resonant frequencies of (a) A_1_ and (b) SH_1_ acoustic waves on the IDT period are presented in Figure 6. It can be seen that in the case of a forward SH_1_ wave, as the IDT period increases, the resonant frequency decreases (Figure 6b). For a backward wave A_1_, an increase in the period of the IDT leads to an increase in the resonant frequency. These dependences behave the same in contact with air and in contact with water.

Thus, as a result of the theoretical and experimental studies carried out, it has been confirmed that the backward acoustic waves do not vanish due to liquid massloading. It opens the possibility to develop new sensor devices based on backward acoustic waves. For this purpose, it is necessary to perform a study of the influence of conductivity and viscosity of liquid on the properties of these waves.

**Figure 7:**
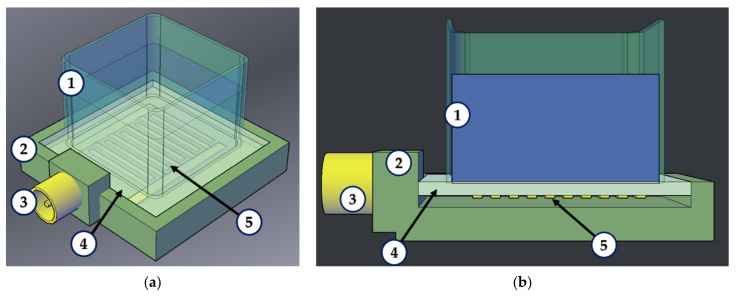
Schematic view of a sensor element based on a resonator on backward acoustic waves. (**a**) general view, (**b**) side view: (1) cell for test liquid, (2) box, (3) coaxial connector, (4) piezoelectric plate, (5) IDT. The possible geometry size is 25 × 18 × 18 mm^3^.

The following scheme can be proposed as a possible design for such a sensor (Figure 7). The piezoelectric plate (4) with the IDT (5) will be placed in a box (2) and fixed with silicone sealant. This will allow isolating the resonator’s IDT (5) from the influence of the liquid measured and minimizing acoustic wave re-reflections from the side faces of the plate and the box. On the surface of the plate free from the IDT, there will be a cell for the test liquid.

The geometry of the structure and the spatial period of the IDT corresponding to the excitation of an acoustic wave with zero group velocity for such a sensor will be defined by using appropriate theoretical and experimental data. As shown in this paper for antisymmetric waves and for shear-horizontal waves [49] in the piezoelectric plates made of lithium niobate and potassium niobate, respectively, the liquid does not lead to a change in the nature of the wave. Thus, it is possible to implement such a device based on backward acoustic waves, including those with zero group velocity. Previously, it was shown that a change in the electrical boundary conditions strongly affects the distribution of the electric and acoustic fields of these waves over the thickness of the plate [32]. This opens the possibility of developing sensors, for example, for the measuring of liquid conductivity. Obviously, the question of the influence of the viscosity and conductivity of the liquid on the characteristics of these waves requires additional research.

## 4. Conclusions

In this paper, a theoretical and experimental study of the influence of a non-viscous and non-conductive liquid on the features of the existence of backward acoustic waves in piezoelectric plates is carried out. It is shown that the presence of liquid on the plate surface leads to a pushing apart between the forward and backward branches of the dispersion dependence of the A_1_ wave, as well as to the appearance of a part of the dispersion curve corresponding to the evanescent wave. One can also see the appearance of the pushing apart between the dispersion dependences for the S_1_ and SH_1_ waves due to the appearance of a liquid load on the plate surface. The results of the experiments have shown that the presence of a non-viscous and non-conductive liquid on the surface of a piezoelectric plate does not change the frequency of the backward acoustic wave but significantly reduces its amplitude. This effect is due to the fact that this wave is characterized by the maximum mechanical displacement component normal to the plate surface. This leads to significant radiation of the mechanical energy of the wave into the liquid. In general, the results obtained confirm the possibility of implementing highly sensitive sensors based on backward acoustic waves in plates. This possibility is due to the proximity of the frequency of existence of these waves to the cutoff frequency and the possibility of excitation of a wave with a zero group velocity. Thus, a slight change in the characteristics of the environment will have a significant impact on the characteristics of this wave. It should be noted that the proposed design of the sensor element will completely isolate the IDT from the environment, and thereby avoid degradation of the electrode structure.

## Figures and Tables

**Table 1:** The material constants of LiNbO_3_ crystal [46].

Elastic Modulii, *C^E^_ij_* (10^10^ N/m^2^)						
*C^E^*_11_	*C^E^*_12_	*C^E^*_13_	*C^E^*_14_	*C^E^*_33_	*C^E^*_44_	*C^E^*_66_
20.3	5.73	7.52	0.85	24.24	5.95	7.28
Piezoconstants, *e_ij_* (C/m^2^)				Dielectric permittivity, *ε^S^_ij_*/*ε*_0_		Density, kg/m^3^
*e*_15_	*e*_22_	*e*_31_	*e*_33_	*ε^S^*_11_	*ε^S^*_33_	*ρ*
3.83	2.37	0.23	1.3	44.3	27.9	4650

## Data Availability

No new data were created or analyzed in this study. Data sharing is not applicable to this article.
